# P-667. The outcomes of Respiratory Syncytial Virus Infection in patients with cancer, solid organ transplant, and other co-morbidities: an international multicenter comparative study

**DOI:** 10.1093/ofid/ofaf695.880

**Published:** 2026-01-11

**Authors:** Tali Shafat, Jonathan Hand, Kamil Manzoor, Nicholas Marschalk, Courtney Nichols, Rotem Gorfinkel, Lior Nesher, David S Y Ong, W Ashwin Mak, Po-Yu Liu, Hsien-Po Huang, Vielka Lopez, Jennifer Jackson, Yusuke Ohashi, Georgios Angelidakis, Amy Spallone, Roy F Chemaly

**Affiliations:** The University of Texas MD Anderson Cancer Center, Houston, Texas; Ochsner Health, New Orleans, LA; Ochsner Medical Center, New Orleans, Louisiana; The Ohio State University Medical Center, Columbus, Ohio; OSU Wexner Medical Center, Columbus, Ohio; Soroka University Medical Center, Beer Sheva, HaDarom, Israel; Soroka Medical Center, Beer Sheva, HaDarom, Israel; Franciscus Hospital, Rotterdam, Zuid-Holland, Netherlands; Franciscus Hospital, Rotterdam, Zuid-Holland, Netherlands; Taichung Veterans General Hospital, Taichung, Taichung, Taiwan; Taichung Veterans General Hospital, Taichung, Taichung, Taiwan; The University of Texas MD Anderson Cancer Center, Houston, Texas; The University of Texas MD Anderson Cancer Center, Houston, Texas; The University of Texas MD Anderson Cancer Center, Houston, Texas; The University of Texas Md Anderson Cancer Center, Houston, Texas; University of Texas MD Anderson Cancer Center, Houston, Texas; The University of Texas MD Anderson Cancer Center, Houston, Texas

## Abstract

**Background:**

Respiratory syncytial virus (RSV) is a common cause of lower respiratory tract illnesses (LRI) worldwide, particularly affecting high-risk groups such as cancer patients, solid organ transplant (SOT) recipients, older adults, and those with significant comorbidities. The Advisory Committee on Immunization Practices (ACIP) recommends vaccinating adults aged ≥75 years, and those aged 60-74 at greater risk for severe RSV disease (1). Recently, they proposed expanding this recommendation to at-risk patients aged 50-59 (2). Our study aims to investigate outcomes of RSV infection and identify risk factors for complications in various populations.

**Table 1:**
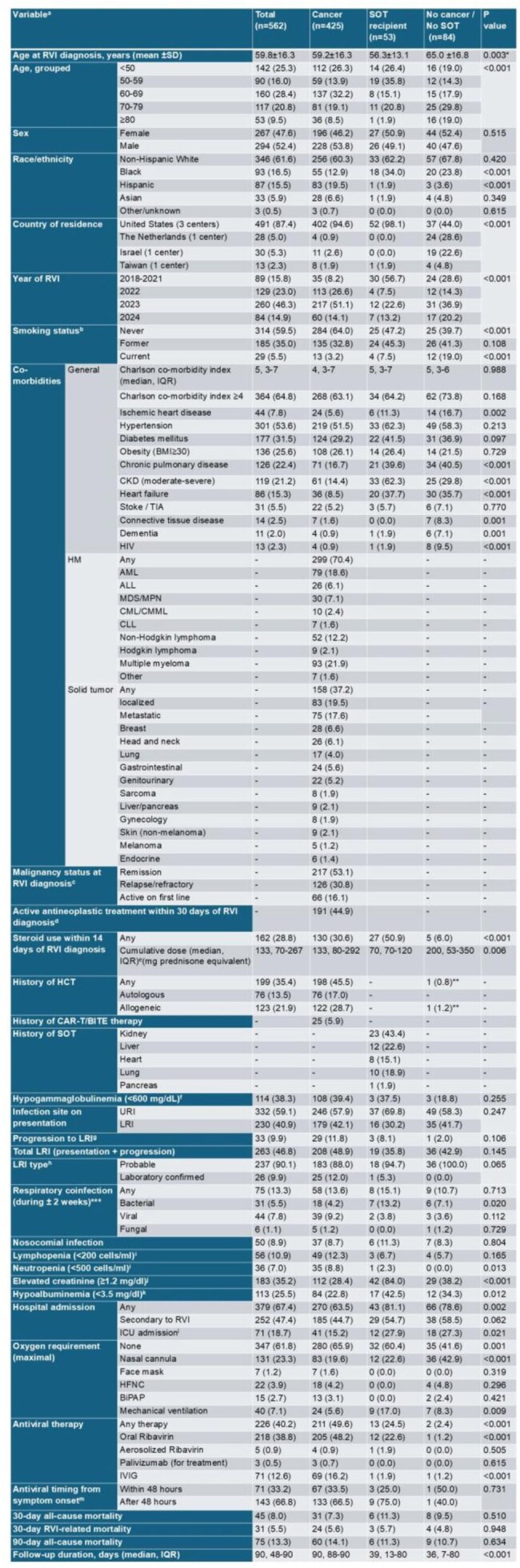
Baseline characteristics and clinical outcomes of patients with RSV-related respiratory viral infection by underlying conditions. aData are no. (%) unless otherwise specified; bn=528; cn=419; dn=435; en=159; fn=298; gn=332; hn=263; in=512; jn=520; kn=443; ln=379=; mn=214. *In post-hoc analysis, the comparisons between the cancer and the non-cancer/SOT groups, and the SOT and non-cancer/SOT groups were statistically significant (p < 0.05). **The HCT indication was primary immunodeficiency disorder. ***Coinfections included rhinovirus (n=18), endemic human coronavirus (n=9), SARS-CoV-2 (n=8), parainfluenza virus (n=3), adenovirus (n=1), influenza virus (n=3), CMV pneumonitis (n=2), Pseudomonas aerogenes (n=10), Streptococcus pneumonia (n=4), Stenotrophomonas melophilia (n=3), Haemophilus influenza (n=3), staphylococcus aureus (MSSA) (n=4), staphylococcus aureus (MRSA) (n=1), Legionella pneumophila (n=1), Nocardia (n=1), Escherichia coli (n=1), Aspergillus (n=4), Zygomycete (n=1), Pneumocystis jirovecii (n=1) . Abbreviations: ALL, acute lymphocytic leukemia; AML, acute myeloid leukemia; BiPAP, bilevel positive airway pressure; BITE, Bispecific T cell Engager; BMI, body mass index; CAR-T, chimeric antigen receptor T-cell therapy; CKD, chronic kidney disease; CLL, chronic lymphocytic leukemia; CMML, Chronic myelomonocytic leukemia; CML, Chronic myeloid leukemia; HCT, hematopoietic stem cell transplantation; HFNC, high-flow nasal cannula; HIV, human immunodeficiency virus; HM, haematologic malignancy; ICU, intensive care unit; IQR, intra-quartile range; IVIG, intravenous immunoglobulin; LRI, lower respiratory tract infection; MDS, myelodysplastic syndrome; MPN, myeloproliferative neoplasms; RSV, respiratory syncytial virus; RVI, respiratory virus infection; SOT, solid organ transplantation; SD, standard deviation; TIA, transient ischemic attack; URI, upper respiratory tract infection.

**Table 2a:**
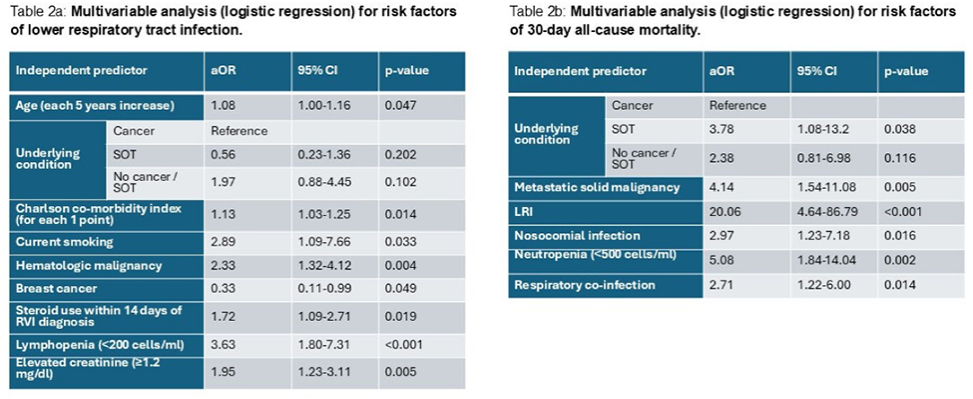
Multivariable analysis (logistic regression) for risk factors of lower respiratory tract infection. Table 2b: Multivariable analysis (logistic regression) for risk factors of 30-day all-cause mortality. Abbreviations: aOR, adjusted odds ratio; LRI, lower respiratory tract infection; RVI, respiratory viral infection; SOT, solid organ transplantation; 95% CI, 95% confidence interval.

**Methods:**

We conducted an international multicenter retrospective observational study including adults with RSV infection across six centers in the USA, the Netherlands, Israel, and Taiwan from October 2018 to April 2024. Patients were categorized based on underlying conditions: cancer, SOT recipients, and those without either. Primary outcomes included LRI and 30-day all-cause and RSV-related mortality.

**Figure 1:**
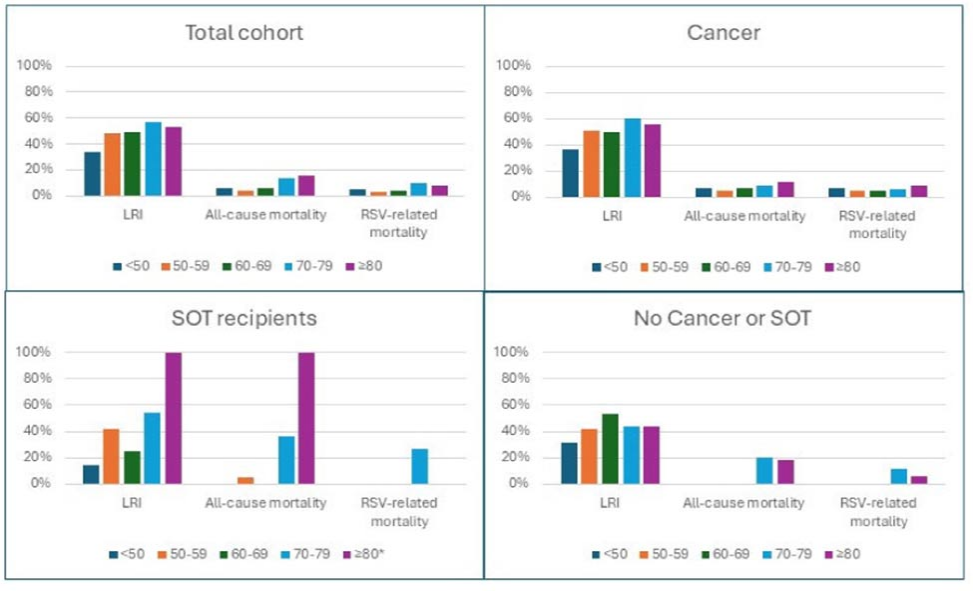
RSV infection outcomes stratified by age groups Abbreviations: LRI, lower respiratory tract infection; RSV, respiratory syncytial virus; SOT, solid organ transplantation. * In the SOT group, only one patient was aged over 80. This patient progressed to LRI and died from another etiology after 19 days of follow-up.

**Figure 2:**
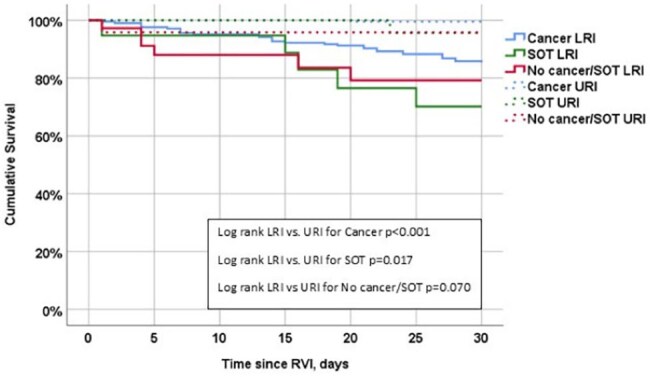
30-day all-cause mortality according to the underlying condition type and the infection site. Abbreviations: LRI, lower respiratory tract infection; RVI, respiratory virus infection; SOT, solid organ transplantation; URI, upper respiratory tract infection

**Results:**

We analyzed 562 patients: 425 (75.6%) with cancer, mainly hematologic malignancies (HMs), and 53 (9.4%) SOT recipients (Table 1). Most patients without either cancer or SOT (84 patients, 15.0%) had significant comorbidities, with 73.8% having a Charlson comorbidity index (CCI) ≥4. Overall LRI rates were 46.8% (48.9% in cancer patients, 35.8% in SOT recipients, and 42.9% in others), with 30-day mortality of 8.0% (7.3% in cancer patients, 11.3% in SOT recipients, and 9.5% in others). LRI and mortality by age group and underlying condition are presented in Figure 1. Independent risk factors for LRI were older age, higher CCI, HMs, smoking, recent steroid use, lymphopenia, and elevated creatinine levels, but the type of underlying condition was not independently associated with LRI (Table 2a). Independent risk factors for 30-day mortality were SOT (compared to cancer), metastatic solid malignancy, nosocomial infection, neutropenia, respiratory co-infection, and LRI (Table 2b, Figure 2).

**Conclusion:**

The study highlights significant morbidity and mortality from RSV in patients with cancer and SOT recipients, as well as in patients without these conditions who are over 50 years of age.

**Disclosures:**

Jonathan Hand, MD, AstraZeneca: Advisor/Consultant|AstraZeneca: Grant/Research Support|Ferring: Grant/Research Support|Innoviva: Advisor/Consultant|Janssen: Grant/Research Support|Pfizer: Advisor/Consultant|Pfizer: Grant/Research Support|Scynexis: Grant/Research Support|The Antibiotic Resistance Leadership Group (ARLG): Grant/Research Support|The Antibiotic Resistance Leadership Group (ARLG): Honoraria Lior Nesher, MD, AstraZeneca: Advisor/Consultant|AstraZeneca: Honoraria|F2G: Grant/Research Support|GSK: Advisor/Consultant|GSK: Honoraria|Medison: Advisor/Consultant|Moderna: Honoraria|MSD: Advisor/Consultant|MSD: Honoraria|Pfizer: Grant/Research Support|Pfizer: Honoraria|Takeda: Honoraria Roy F. Chemaly, MD, MPH, FIDSA, FACP, FESCMID, ADMA Biologics: Advisor/Consultant|AiCuris: Advisor/Consultant|AiCuris: Grant/Research Support|Ansun Biopharma: Advisor/Consultant|Ansun Biopharma: Grant/Research Support|Assembly Bio: Advisor/Consultant|Astellas: Advisor/Consultant|Eurofins Viracor: Advisor/Consultant|Eurofins Viracor: Grant/Research Support|Genentech: Grant/Research Support|Gilead: Advisor/Consultant|InflaRX: Advisor/Consultant|IntegerBio: Advisor/Consultant|Karius: Advisor/Consultant|Karius: Grant/Research Support|Merck/MSD: Advisor/Consultant|Merck/MSD: Grant/Research Support|Moderna: Advisor/Consultant|Oxford Immunotec: Advisor/Consultant|Pfizer: Advisor/Consultant|Shionogi: Advisor/Consultant|Takeda: Advisor/Consultant|Takeda: Grant/Research Support|Tether: Advisor/Consultant

